# Influence of Metallic Deposition on Ceramic Femoral Heads on the Wear Behavior of Artificial Hip Joints: A Simulator Study

**DOI:** 10.3390/ma13163569

**Published:** 2020-08-12

**Authors:** Jessica Hembus, Lisa Rößler, Mario Jackszis, Annett Klinder, Rainer Bader, Carmen Zietz

**Affiliations:** Biomechanics and Implant Technology Research Laboratory, Department of Orthopaedics, University Medicine Rostock, Doberaner Str. 142, 18057 Rostock, Germany; lisa.roessler@uni-rostock.de (L.R.); mario.jackszis@med.uni-rostock.de (M.J.); annett.klinder@med.uni-rostock.de (A.K.); rainer.bader@med.uni-rostock.de (R.B.); carmen.zietz@innoproof.de (C.Z.)

**Keywords:** retrievals, hip wear simulator, total hip replacement, third-body wear, metallic deposition, metallic transfer, ceramic head

## Abstract

Several retrieval studies have reported on metallic depositions on ceramic femoral heads, but the effect on the wear behavior of artificial hip joints has not been investigated in wear simulator studies. In the present study, retrieved ceramic heads with metallic depositions as third particles were tested against cross-linked ultra-high-molecular-weight polyethylene (UHMWPE) liners in a hip wear simulator. The amount of liner wear and expansion of metallic depositions on the heads were determined before and after wear testing with digital microscopy. The surface roughness of the heads was investigated in areas with and without metallic depositions by laser scanning microscopy. After five million load cycles, a non-significant reduction in the metallic formation on the retrieved heads was found. The metallic areas showed a higher surface roughness compared to unconcerned areas. The liners showed a higher wear rate of 1.57 ± 1.36 mg/million cycles for 28 mm heads and 2.42 ± 0.82 mg/million cycles for 36 mm heads with metallic depositions, in comparison with new ceramic heads with a 28 mm size ((−0.06 ± 0.89) mg/million cycles) and 36 mm size ((2.04 ± 0.46) mg/million cycles). Metallic transfer on ceramic heads can lead to an increased surface roughness and higher wear rates at the UHMWPE liners. Therefore, metallic contact of the ceramic femoral head should be avoided.

## 1. Introduction

The main cause of total hip revision is aseptic loosening caused by wear particles [1,2]. In order to increase the durability of artificial hip joints, the amount of wear debris has to be minimized and the tribological properties of the articulating surfaces have to be optimized [3]. Tribological properties of ceramic bearings have been proven to be advantageous over metal bearings [4,5]. Therefore, the ceramic femoral head has become a low-friction standard material [6,7,8,9] and combined with polyethylene (PE) as a bearing couple, it is an established low-abrasion bearing in total hip replacement [9,10].

In retrieval studies of ceramic femoral heads, authors have reported dark shiny metallic formations on the surface, mainly at the equator of the head [2,3,11,12], due to the transfer of metallic material to the surface of the ceramic head [11,12,13,14,15]. Such formations were also described in a simulator study during the testing of ceramic-on-metal bearings [13,16] and they can appear linear and planar. The metallic transfer can occur in the smallest contact area of the ceramic head with the rim of the metallic cup [3,17,18]. According to Luchetti et al. [11], these effects can result from total hip subluxation or dislocation, since this area is within the articulating surfaces during a normal gait. Most studies indicate that metallic transfer is caused by the malpositioning, loosening, or dislocation of implant components [3,6,17]. Dorlot et al. [17] found an average area of 67 mm^2^ (5–850 mm^2^) for metallic transfer on the retrieved ceramic heads. In a recent study, metallic transfer areas in the same size range were found [15]. A correlation between the extent of metallic transfer and the age, sex, weight, and activity of patients could not be detected [3,17]. Furthermore, an increase in the surface roughness in the areas with metallic depositions was reported [3,8]. An increased surface roughness of the femoral head led to enhanced polyethylene wear of the liner and to elevated third-body wear [3,8,11,13,15,19]. In a retrieval study by Kim et al. [3], higher wear of the polyethylene liner with increasing contamination of the heads was found. So far, research on metallic depositions on ceramic heads has only focused on macroscopic and microscopic analyses of retrievals.

The aim of the present study was to determine the influence of metallic depositions on ceramic femoral heads on the wear behavior of ceramic-on-PE bearings under standardized test conditions using a hip wear simulator. Several clinical studies have reported an increase in wear by metallic transfer on the femoral head [3,11]. Experimental investigations using ceramic-metal bearings also showed metallic depositions on the femoral component [13,16]. Nevertheless, standard wear test setups with ceramic-on-PE bearings with metallic depositions have not been performed thus far. In addition, the influence of metallic transfer on different head sizes was determined. The data were compared to the results of a previous study using new ceramic-on-PE bearings with an identical design.

## 2. Materials and Methods

### 2.1. Test Specimens

**Figure 1:**
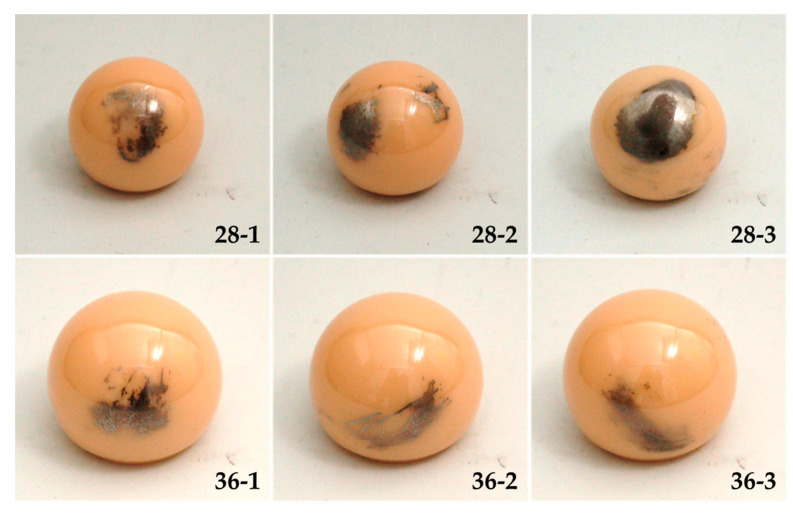
Retrieved femoral heads made of alumina (Al_2_O_3_) with metallic transfer, 28 mm (*n* = 3) and 36 mm (*n* = 3) in diameter.

Retrieved alumina femoral heads with shiny metallic areas were used (Figure 1) for a hip wear simulator test. Three femoral heads that were 28 mm in diameter and three femoral heads that were 36 mm in diameter, made of alumina (Al_2_O_3_) with metallic markings, were selected. In order to ensure comparable ceramic femoral heads for the study, heads with similar distributions and areas of metallic depositions were selected from the retrieval archive of our hospital. The retrieved ceramic heads were part of uncemented implant systems combined with polyethylene liners used as a bearing partner. The implantation period of the 28 mm heads was between 303 and 4769 days and between 51 and 192 days for the 36 mm heads. For the hip wear simulator, the selected retrieved ceramic heads (Figure 1) were combined with new cross-linked ultra-high-molecular-weight polyethylene liners in combination with uncemented acetabular cups (Trident X3, Stryker GmbH & Co. KG, Duisburg, Germany) with an outer diameter of 56 mm. The study was approved by the Ethics Committee of the University of Rostock (registration number A 2017-0141).

### 2.2. Hip Simulator Test and Wear Measurement

In order to investigate the influence of metallic transfer on the wear rate of ceramic-on-PE bearings, a wear test using a standard hip wear simulator according to ISO 14241-1 [20] was performed. For saturation, the PE liners were stored in the test fluid until saturation for eight weeks at room temperature. Bovine serum (Biochrom GmbH, Berlin, Germany) with a protein content of 30 g/L, including 7.44 g/L ethylene diamine tetra acetic acid (EDTA) and 1.85 g/L sodium acid (NaN3, 0.2%), was used as the test fluid.

For wear simulation, the selected retrieved heads (Figure 1) were aligned, so that the surfaces with metallic depositions were in the articulating area and, consequently, in the mainly loaded area. The wear test was conducted in a hip wear simulator (Endolab GmbH, Rosenheim, Germany), in accordance with ISO 14242-1 [20]. Three dynamic loaded stations and one axial loaded soak control to validate fluid absorption were used for each head size (28 and 36 mm). Since the soak control does not produce any abrasion, the weight change of the PE liners can be directly linked to the amount of fluid absorption. By subtracting the fluid adsorption from the mass change of the dynamically loaded liners, the abrasion was determined. The measured wear data are shown as the average and standard deviation.

In the hip wear simulator, the tested specimens were loaded according to the movements and forces of a normal gait defined by ISO 14242-1 [20]. This includes an axial load of between 0.3 and 3 kN and movements between 18° and 25° extension/flexion, −4° and 7° abduction/adduction, and −10° and 2° external/internal rotation. The movements and loads of the bearing surfaces were applied in isolated and tempered with (37 ± 2) °C test chambers with 1 Hz for five million cycles. Every 0.5 million cycles, the bovine lubricant was changed and the weight of the liners was measured gravimetrically using a high-precision balance (Sartorius ME235S, Sartorius AG, Göttingen, Germany, sensibility 0.01 mg, uncertainty 0.03 mg), in accordance with ISO 14242-2 [21]. In order to exclude station-conditioned influences, the test implants were changed periodically between the running stations. The measured wear data were compared to the wear rates from our previous studies [22,23].

### 2.3. Measurement of the Expansion of the Metallic Area

The total areas of the metallic deposition of the alumina femoral heads used in the hip wear simulator (Figure 1) were measured with a digital microscope before and after the wear test in the hip simulator (VHX-900F, Keyence Germany GmbH, Neu-Isenburg, Germany) using 3D images of the surfaces. The recorded values were compared after the abrasive wear test.

### 2.4. Analysis of Surface Roughness

The roughness measurements were performed for all femoral heads shown in Figure 1 after five million cycles in the hip wear simulator. The maximum height (Rz) and arithmetic average roughness (Ra) were determined optically with a laser scanning microscope (LSM, VK-X250, Keyence Germany GmbH, Neu-Isenburg, Germany), according to DIN EN ISO 3274: 1998 [24] and DIN ES ISO 4288: 1998 [25]. For comparison, the roughness of each ceramic head was examined at three different locations on the unaltered surface without visible metallic deposition and at three areas with metallic depositions on the same retrieved head. Four roughness measurements were obtained for each location and the roughness values of the unaltered and altered surface were compared.

### 2.5. Statistical Analysis

Statistical analysis was performed using IBM^®^ SPSS^®^ software (Statistics version 20, IBM Corporation, Armonk, NY, USA). The Gaussian distribution of the values for PE wear rates with new ceramic heads and heads with metallic depositions, the metallic transfer area on the femoral heads before and after loading in the hip wear simulator, and the roughness of the head surface with and without metallic depositions were analyzed with the Kolmogorov–Smirnov test. For statistical comparisons, *P*-values of <0.05 were considered significant. The statistical tests performed are explained in further detail in the respective result section.

## 3. Results

### 3.1. Wear Rates

**Figure 2:**
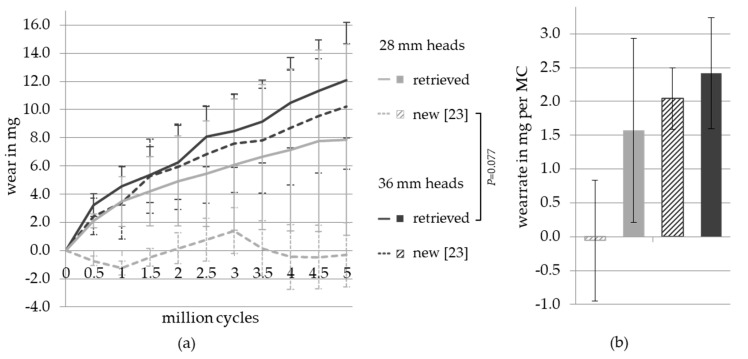
Mean gravimetric wear of the sequentially cross-linked polyethylene (PE) liners combined with new [23] and retrieved 28 and 36 mm alumina femoral heads. (**a**) Total wear over five million cycles and (**b**) wear rates per million cycles.

The gravimetric wear of the PE liners combined with new and retrieved ceramic femoral heads with metallic transfer (28 and 36 mm diameters) is shown in Figure 2. The graphs show an almost linear increase in wear over five million cycles (Figure 2a), whereby the wear of the liners articulated against the retrieved femoral heads (36 mm head size) was the highest across all five million cycles (MC), with a total wear of (12.09 ± 4.12) mg ((2.42 ± 0.82) mg per MC). This was followed by liners articulated against the new femoral heads (36 mm head size), with a total wear of (10.21 ± 2.28) mg ((2.04 ± 0.46) mg per MC) and by liners articulated against retrieved 28 mm femoral heads with metallic depositions, with a total wear of (7.86 ± 6.79) mg ((1.57 ± 1.36) mg per MC). The combination of PE liners with new 28 mm femoral heads from the standard test showed nearly no change in gravimetric weight, with a total wear of (−0.29 ± 4.45) mg ((−0.06 ± 0.89) mg per MC). For determination of all wear rates, the zero value was included.

Figure 2b shows the wear rates of the four bearing couples per MC. The wear values were not normally distributed. The standard test surface was compared to the metallic deposition surface by a two-way analysis of variance (ANOVA), with the head size and surface area as variables. The wear values of the PE liners, articulated against the 36 mm femoral heads, were significantly higher than the wear values of the bearings with the 28 mm femoral heads (*p* = 0.047). The difference between the wear values of the new and retrieved femoral ceramic heads with metallic depositions was not statistically significant (*p* = 0.150). Moreover, the interaction between the variables head size and condition of femoral head (new or retrieved) showed no significant influence on wear rates (*p* = 0.350).

The change in abrasive wear over time shown in Figure 2a was statistically analyzed for all four groups in a Repeated Measures two-way ANOVA (mixed model) with Tukey’s post hoc test and Greenhouse–Geisser correction. The analyzed variables were the time and different groups. The increase in wear values over time was significant (*p* = 0.001). When comparing all groups, there was a trend for groups differing from each other (*p* = 0.079). The highest difference was found between the new femoral heads with a diameter of 28 mm and the retrieved heads with a diameter of 36 mm (*p* = 0.077).

In summary, Figure 2 shows that the PE liners articulated against heads with metallic depositions tended to exhibit higher wear. The distinctly higher standard deviations in bearings with metallic transfer probably contributed to the fact that the observed differences did not reach significance.

### 3.2. Measurement of the Expansion of the Metallic Area

**Figure 3:**
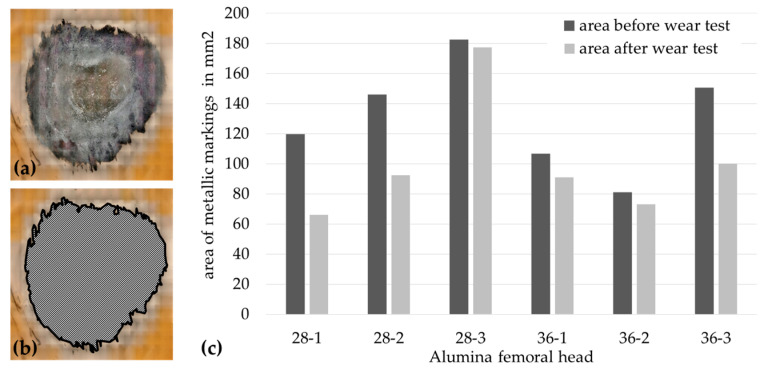
Measurement of metallic deposition areas. (**a**) Metallic depositions, (**b**) marked area for evaluation, and (**c**) recorded values for metallic depositions on retrieved alumina ceramic heads.

The area of metallic depositions was determined on all six femoral ceramic heads before and after the hip simulator test. The measured values were normally distributed. For comparisons of the area before and after the wear test, an unpaired t-test was used. The recorded areas are shown in Figure 3. Before wear simulator testing, the average area of the metallic depositions for all heads was (131.09 ± 35.93) mm^2^ (81.16 mm²–182.45 mm^2^). After the wear test, the average area decreased to (100.08 ± 40.01) mm^2^ (65.99 mm^2^–177.41 mm^2^). The total area of metallic depositions on the femoral heads before and after the articulation of bearings in the wear simulator was not significantly different (*p* = 0.188). However, Figure 3 shows a slightly reduced metallic deposition area after articulation of the surfaces. The difference was particularly evident in femoral heads 28-1 and 28-2.

### 3.3. Analysis of Surface Roughness

Since the head size did not significantly influence the roughness Ra (*p* = 0.643) and the maximum height Rz (*p* = 0.689), the comparison of the original surface and metal transfer was based on all six retrieved heads. The Mann–Whitney U test was used for statistical evaluation, as the recorded roughness values for Ra and Rz did not show a Gaussian distribution.

**Figure 4:**
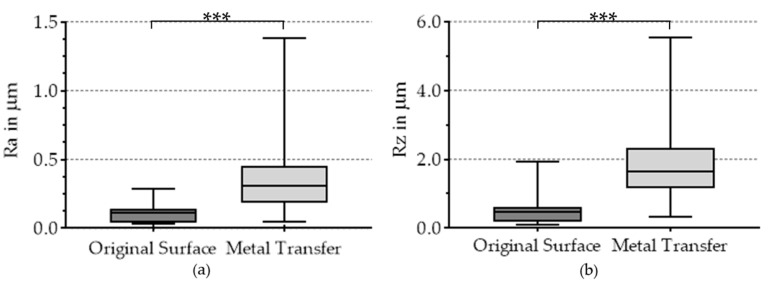
Results of the surface analyses of the ceramic heads with (**a**) arithmetic average roughness Ra and (**b**) maximum height Rz; highly significant differences marked with three asterisks.

Surfaces with metallic depositions displayed significantly higher (*p* < 0.001) Ra (mean ± SD: (0.35 ± 0.23) μm) than the unaltered surfaces of the retrieved femoral heads (mean ± SD: (0.09 ± 0.06) μm). Rz was also significantly higher (*p* < 0.001) in the areas with metallic depositions (mean ± SD: (1.90 ± 1.04) μm) than on the unaltered surface of the retrievals (mean ± SD: (0.49 ± 0.42) μm). The distribution of the roughness parameters is shown in Figure 4.

## 4. Discussion

In order to investigate the influence of metallic deposition, a wear test using a standard hip wear simulator according to ISO 14241-1 [20] was performed in the present study. The physiological conditions in the human hip joint cannot be completely simulated by such standard tests; however, for a comparison of different bearings, the wear test method with standardized testing conditions has been successfully established and validated [26]. The same applies to bovine serum used as a norm-compliant lubricant for the standard wear testing of artificial joints.

The area of the metallic depositions was measured with a digital microscope. It should be noted that the measurements could be affected by reflections on the smooth surface. This undesirable effect was largely minimized by the use of diffusers. Nevertheless, the smallest metallic deposition could not be included in the survey of the transferred areas, and only the areas around the main articulated area were included. Another limitation was the selection of the femoral ceramic heads. Although care was taken to use heads with the same amount of metallic contamination, not all ceramic heads showed the same expansion or thickness distribution of metallic depositions. Furthermore, femoral heads from left and right total hips were used. The influence should be minimized by aligning the metallic depositions in the mainly loaded area. To obtain complete comparability, wear simulations with unused ceramic heads after standardized metallic deposition might represent a suitable alternative. Since the material composition of the metallic deposition was not known, this should be determined in further investigations by means of energy-dispersive X-ray spectroscopy (EDX). The measured extent of the metallic depositions was similar to the extent already described by Dorlot et al. [17], which ranged from 5 to 850 mm^2^, and by Affatato et al. [15], which ranged from 29.6 to 573.6 mm^2^, on ceramic heads. The measured areas on the heads before and after wear testing are not significantly different. However, the statistical significance of this test is only partially meaningful, because the thickness or volume of the metallic depositions could not be measured in this study. Furthermore, the non-significant decrease in the deposition area indicates that the removal of the metallic volume could have a significant influence on the wear rate. The reduction of metallic depositions was apparent (Figure 3), especially for heads 28-1 and 28-2. One explanation for the difference could be the thinner metallic deposit on both heads as opposed to head 28-3. A reduction in the deposited metallic amount on the head surface causes the release of metallic depositions in the form of metal particles and metal ions, which indicates the presence of third-body particles in the joint space and surrounding tissue. The correlation between the volume deviation of the metallic depositions and the wear rate of the polyethylene liner may confirm this relationship. Furthermore, the thickness and extent of the metallic dispositions may depend on the lifetime of the implant in situ.

For roughness measurements on the retrievals, an LSM was used. Four perpendicular roughness measurements per analyzed area were obtained in order to exclude the influence of one directional pattern on the determined roughness parameters. Significantly higher roughness values for Ra and Rz were detected in the areas with metallic depositions than on the unaltered ceramic surface. Thereby, high standard deviations in roughness values for the metallic deposition were noted. This is probably due to the different deposition thicknesses, the different materials, or the residence time of the implant before retrieval. A limitation is the roughness measurement on the unaltered surface, since the measurement of invisible thin metallic deposits cannot be excluded. It should also be noted that the roughness of the bearing surfaces in general increases with the retention time in the human body. The measured roughness values for Ra were similar to measured data on ceramic femoral heads from Affatato et al. [15], who determined a value of (0.3 ± 0.1) µm in areas with metallic deposition and (0.03 ± 0.1) µm on unaltered surfaces.

By aligning the areas with metallic deposition in the mainly loaded articulation surface, the influence of the metallic deposition should be increased as much as possible to simulate a worst-case scenario. Nevertheless, no significant differences in wear rates of ceramic-on-PE bearings with and without metallic deposition could be determined. In Figure 2, the total wear over five million cycles, as well as the wear rate per million cycles, show a slightly higher wear of PE on heads with metallic deposition than on the new heads. As described, the size of the femoral heads exhibited a significant influence on the wear rate of PE liners in the present study. It is well-known that larger femoral heads lead to a higher wear rate of the bearing [22,23,27] due to an increased sliding distance, reduced contact pressure with larger heads [28], and different clearances [29]. It should be emphasized that the bearings of the 28 mm heads with metallic deposition produced significant wear, which was almost in the order of the wear rates of the bearings with 36 mm heads, while in the standard test with new 28 mm ceramic heads, nearly no gravimetric wear could be measured [23]. Zietz et al. [23] found a higher level of fluid absorption than gravimetric wear for alumina-on-PE bearings with a 28 mm head size. Yan et al. [30] showed that wear rates decreased after the end of a running-in phase and the beginning of the steady state phase; thus, the zero point should be excluded from the calculation. This is also required by ISO 14242-2 [21]. However, in order to ensure comparability with the study of Zietz et al. [23], the zero point was also taken into account in the calculation of wear rates in this study. Since the same hip wear simulator was used in both studies, the inclusion of the zero point presents no disadvantage. Another point to consider is the high standard deviations in the wear data of the PE liners. These may be due to the different extents and thicknesses of the metallic depositions on the retrieved femoral heads. Another possible reason may be the different ablation of the metallic film under cyclic loading of the heads. This would be an indication that the increased wear rates are caused by third-body particles released from the depositions. However, since similar high standard deviations occurred in the comparative study of Zietz et al. [23], the test appears to be very robust and comparable, even with the low number of samples. An important limitation of our study was that the wear rate of retrievals with metallic deposition was not compared to retrievals without metallic deposition, and only with new femoral ceramic heads. However, in the study of Kim et al. [3], no significant differences in surface roughness between retrieved and new ceramic femoral heads could be detected. Furthermore, the same new PE liners were used in combination with both the new and retrieved femoral ceramic heads, and nearly linear wear over five million cycles was achieved. Even though the exact influence of metallic deposition could not be determined, the dynamic load of normal walking still leads to abrasive wear of the metallic deposition. According to a study by Kim et al. [3], the surface roughness increases with the grade of contamination by metallic deposition, which leads to an increase in the wear rate. Due to the movement and friction on the metallic deposition, metallic particles are released from the surface and reach the joint space, where they may lead to increased wear, acting as third bodies. The increase in wear rates by third-body particles has already been investigated and confirmed in several studies [19,31,32]. Therefore, metallic depositions, for instance, caused by subluxation, dislocation, and reposition of the artificial hip joint or any contact of the ceramic components with metallic components intraoperatively and postoperatively, should be avoided [2,7,17]. Moreover, in total hip revision, ceramic heads with metallic depositions should be exchanged.

According to Kim et al. and Dorlot et al., patient history has no influence on the extent of metallic transfer, but a link between undergoing total hip revision and the extent of metal transfer cannot be excluded [3,17]. Luchetti et al. [11] suggested that metallic transfer on femoral cobalt-chromium (CoCr) heads cannot be excluded. Therefore, further retrieval studies on metallic transfer on femoral heads are needed. Investigational studies of metallic transfer on the retrieved ceramic heads for determination of the material composition are currently planned. This may clarify the origin of the metallic deposition, which can be derived from the acetabular cup after subluxation or dislocation, surgical instruments, screws, or other implant materials. The most commonly expected materials are titanium, cobalt-chromium, and stainless steel, since these are often used as implant materials or surgical instruments [11,12,13]. Since the metallic depositions occurred in femoral heads with an implantation period of only 51 days (femoral head 36-1, see Figure 1), the probability is high that the metal application was caused during implantation. The reason for the retrieval was not recorded and could not be discussed in this study.

Furthermore, an analysis of wear particles from the bovine lubricant of the simulator study could provide detailed information on the origin and shape of the third-body particles from the metallic transfer, as well as their abrasive behavior.

## 5. Conclusion

When studying the influence of metallic depositions on ceramic femoral heads on PE inserts using a hip wear simulator, heads with metallic depositions exhibited a significantly increased surface roughness and led to an increase of wear rates compared to new ceramic femoral heads. Therefore, metallic depositions, for instance, caused by subluxation, dislocation, and reposition of the artificial hip joint or any contact of the ceramic components with metallic components intraoperatively and postoperatively, should be avoided. Moreover, in total hip revision, ceramic heads should be exchanged, as metallic deposition cannot be ruled out.
